# Cohort Records Study of 19,655 Women Who Received Postabortion Care in a Tertiary Hospital 2010–2013 in China: What Trends Can Be Observed?

**DOI:** 10.1155/2016/3629451

**Published:** 2016-02-18

**Authors:** Zhen-Yu Luo, Song Quan, Dong-Ning Chai, Wei-Hong Zhang

**Affiliations:** ^1^Center for Reproductive Medicine, Nanfang Hospital, Southern Medical University, Guangzhou 510515, China; ^2^Family Planning Clinic, Xiamen Maternity and Child Health Care Hospital, Xiamen 361003, China; ^3^School of Public Health, Université libre de Bruxelles (ULB), 1070 Brussels, Belgium

## Abstract

The retrospective cohort epidemiological study was to investigate the characteristics of women who underwent induced abortion. Data were retrospectively collected from women who underwent induced abortions (*n* = 19,655) at the Xiamen Maternity and Child Health Care Hospital (2010–2013). The characteristics of women who underwent induced abortions included mean age, unmarried status, no previous deliveries, first pregnancy, ≥2 abortions including the current one, and a history of caesarian section. From 2010 to 2013, mean age increased and declines were observed in the ratio of induced abortions to live births, the proportion of induced abortions among women of 15–24 years, those who were unmarried, had their first pregnancy, or had no history of delivery. However, the rates of induced abortions increased among women who were lactating, had a history of caesarian section, or had an interpregnancy interval of <6 months. This snapshot of induced abortions in China might suggest that the numbers are increasing but the ratio to live births has fallen. Methods should be improved to prevent unwanted pregnancies and reduce the number of induced abortions in China. It must be emphasized that differences in mentality and culture between countries might limit the representativeness of these results.

## 1. Introduction

An induced abortion is a global public health and social issue, and governments have made extensive efforts to reduce the rates of induced abortion. In 2003, the World Health Organization (WHO) produced a handbook entitled* Safe Abortion: Technical and Policy Guidance for Health Systems* to guide the planning and delivery of safe abortion services [1]. In addition, studies have demonstrated that providing contraceptive counseling as well as services during postabortion care (PAC) visits is critical factor for reducing the subsequent number of induced abortions [2]. 

In 2011, Chinese Medical Association released a Post-Abortion Family Planning Service Guide and created a special nonprofit fund to help hospitals in establishing a PAC clinic (the YIAI fund). These PAC clinics reduce the rates of induced abortion by providing PAC services for all women who have had induced abortions. As women who undergo induced abortion at >10 weeks of gestation have to be hospitalized for their safety, women may only terminate a pregnancy at a PAC clinic if they are at ≤10 weeks of gestation [1, 2].

The current sociodemographic knowledge about women who have undergone induced abortions in China is limited by studies with a relatively small sample size or a lack of updated information. Therefore, the objective of this study was to characterize and analyze the epidemiological trends among women who underwent induced abortion in the safe setting of a tertiary specialized center and who subsequently received PAC services at this single institution. This data might provide a snapshot of the current trends for induced abortion in China just prior to the recent easing of the one-child policy that allows couples to have a second child. This study might provide evidence regarding how to decrease the rate of induced abortion and improve women's reproductive health.

## 2. Materials and Methods

This was a retrospective cohort epidemiological study of women who underwent induced abortions between 2010 and 2013 at the Family Planning Clinic, Xiamen Maternity and Child Health Care Hospital, China. This hospital is a tertiary teaching hospital, has a high volume of patients, and performs approximately 12,000 deliveries each year.

Therefore, the inclusion criteria were as follows: (1) the pregnant women terminated pregnancy voluntarily without contraindications; (2) the women were at ≤10 weeks of gestation; (3) the intrauterine pregnancy was confirmed by B-mode ultrasound examination; and (4) the induced abortion operations were performed at the outpatient department. The exclusion criteria were as follows: (1) abortion undertaken for medical reasons (such as missed abortion) or (2) women with incomplete records.

A data extraction sheet was designed to extract the required information (i.e., date, age, marital status, number of pregnancies, number of deliveries, number of previous abortions, and high risk factors) from the records provided for the induced abortion procedure of the women who underwent an abortion at the Family Planning Clinic. The reasons for unwanted pregnancies are collected in the medical charts as a standard practice at our center in order to provide subsequent tailored psychological PAC, if needed. Patients whose medical records had missing or incomplete information were excluded from the study (*n* = 9).

The study investigators all had a medical background (i.e., doctors and high degree nurses), and all investigators were trained to use the extraction sheet. Data from the patients' medical records were extracted anonymously and were only used for the present study. Ethical approval was obtained from the institutional review board.

Induced abortions of pregnancies with intrauterine device (IUD)* in situ* and abortion during the first half year after delivery and lactation period and longitudinal diaphragmatic uterus were considered as high risk.

The collected data were exported and analyzed using SPSS 16.0 (IBM, Armonk, NY, USA). Categorical data are presented as frequencies and percentages. Continuous data are presented as means and standard deviations. A trend analysis was used to identify the independent factors that were related to the incidence of induced abortions that were conducted between 2010 and 2013 at our facility. Differences with a *P* value <0.05 were considered statistically significant.

A tendency analysis was also conducted regarding age, marital status, number of pregnancies, and number of deliveries of women who underwent induced abortion, to observe the changes over the time course from 2010 to 2013.

## 3. Results

### 3.1. Characteristics of the Abortions

Most women were undergoing their first induced abortion (*n* = 9300, 47.3%), although 6173 (31.4%) were undergoing their second abortion, and 4182 (21.3%) women were undergoing at least a third abortion; the highest number of abortions observed for a single patient was 15 (Table 1).

### 3.2. High Risk Abortions

Among the 19,655 women in this study, 959 (4.9%) women underwent high risk abortions; these included 349 (1.8%) women with IUD, 159 (0.8%) lactating women, 391 (2.0%) women with an interpregnancy interval of <6 months, and 77 (0.4%) women with uterine malformation (Table 1).

### 3.3. Basic Demographic Characteristics

Mean age at abortion was 27 ± 6 years (range, 15–48 years). Among the 19,655 women, most were 20–29 years old (*n* = 12,658, 64.4%) and 891 (4.5%) were <20 years old. In addition, 7846 (39.9%) women were unmarried, 5803 (29.5%) were experiencing their first pregnancy, 9797 (49.8%) were nulliparous, and 2576 (13.1%) had a history of caesarian section (Table 1).

### 3.4. Reduction in the Ratio of Induced Abortions to Live Births between 2010 and 2013

The ratio of induced abortions to live births was sensitive to the changing abortion trends over the 4-year period (Table 2). The total number of induced abortions increased from 3906 in 2010 to 5403 in 2013, while the ratio of induced abortions to live births underwent a steady decline from 48.8% in 2010 to 42.3% in 2013 (*P* < 0.01).

### 3.5. Unwanted Pregnancies

Among all 19,655 women, 6958 (35.4%) did not use any contraception at all; 5916 (30.1%) were usually having sex during the safe period; 2889 (14.7%) had to use emergency contraception; 2142 (10.9%) were using coitus interruptus; 1228 (6.2%) were using condoms; 349 (1.8%) were using IUDs; and 173 (0.9%) were using other methods (diaphragm, contraceptive patch, etc.).

### 3.6. Reduction in Induced Abortions among Younger Women and Unmarried Women, for First Pregnancy and for Nulliparous Women

The average age increased from 27 ± 0.09 years in 2010 to 28 ± 0.07 years in 2013. Among women receiving abortions, 42.5% were 15–24 years old in 2010; in 2013, that proportion decreased to 33.1%. Similarly, induced abortions among unmarried women decreased every year, from 44.8% in 2010 to 36.1% in 2013 (*P* < 0.01). Among women who underwent an abortion at our facility, the proportion of women who were experiencing their first pregnancy declined during the 4-year period (Table 3). Among women without a history of delivery, the frequency of induced abortions decreased from 56.35% in 2010 to 45.2% in 2013.

### 3.7. Increase in Abortions in Older and in Married Women

The frequency of induced abortions at our facility among women who were 25–48 years old and among married women increased during the 4-year period (Table 3).

### 3.8. Trends in High Risk Abortions

In the PAC hospital population, the frequency of induced abortions for women with a history of caesarian section increased from 10% to 18% over the 3-year period (*P* < 0.1) (Table 3). Among the 19,655 women, an increasing trend in the frequency of induced abortions was observed during the 4-year period among women who were lactating (from 0.59% to 1.02%) or who had an interpregnancy interval of <6 months (1.09% to 2.79%). Furthermore, a gradual decreasing trend in the rate of induced abortions was observed among women with IUDs* in situ*, from 2.20% in 2010 to 1.45% in 2012, although 1.67% of women experienced IUD failure in 2013. Similarly, the frequency of induced abortions among women with uterine malformations decreased from 0.44% in 2010 to 0.24% in 2012, although it subsequently increased to 0.57% in 2013.

## 4. Discussion

In this study, among the 19,655 women who underwent an abortion at our facility, the percentage of women who were <25 years old, were experiencing their first pregnancy, or had repeated abortions (including the current pregnancy) was 37.5%, 29.5%, and 52.7%, respectively. In a recent report regarding induced abortions in China (2010), 47.5% of the women were <25 years old and 35.8% were experiencing their first pregnancies [3], which supports this present study. Similar results were also observed in the United States [4]. These may reflect several trends regarding induced abortion in the general Chinese population.

In this study, although the number of induced abortions increased in time, the decreased ratio of abortions to live births suggests that this is different to the stalled decline in abortion rates worldwide that has remained relatively static after a decline from 1995 to 2003 [5]. However, in developing countries, abortion rates vary dramatically and many countries do not provide full data [6].

In this study, although most of the indexes decreased over the 4-year study period, the rate of induced abortions increased in three categories: those who were lactating, those whose interpregnancy interval was <6 months, and those who had a history of caesarian section. In each of these situations, it could be considered that the reasons for choosing abortion by these women were similar to those of women worldwide [5, 7, 8]. This might be suggested by other studies in Ghana and Pakistan that show an increased number of induced abortions in women who have already had a previous pregnancy/children, but the detailed reasons and time between pregnancies are not given in those previous studies [9, 10]. For women with a history of caesarian section, other reasons that may influence the decision include women still recovering from the procedure, women who did not wish to risk another difficult birth, or women who considered that one, two, or three caesarian sections were enough to complete a family [11]. Therefore, this increase in abortion number observed in this study might also be related to the high rate of caesarian section in China. Although WHO population-based survey found that the global rate of caesarian section was 27.3%, China had the highest rate (46.2%) [12]. The ratio of induced abortions with high risk factors to live births increased annually. Currently, there is lack of data on induced abortions in women with high risk factors. These abortions have to be examined in future studies.

Similar to previous Chinese studies [13, 14], the absence or poor use of contraceptives was the main cause of unplanned pregnancies in this study, followed by failure of withdrawal, timing, and emergency contraceptive methods. In addition, most lactating women who underwent abortions believed that they would not become pregnant immediately after delivery without experiencing their menses, as has been previously reported [15]. This may explain why the frequency of induced abortions increased among the women who had an interpregnancy interval of <6 months. As a previous study reported, condoms are the most commonly used contraceptives [15]. However, many women are unlikely to use short-acting oral contraceptives or IUDs, as they dislike taking a pill every day, worried about the various side effects, and/or believe that these contraceptives can harm their overall health [16–18]. The main strength of this present study is the large number of included women (*n* = 19,655) and the very small number of excluded women (*n* = 9). Nevertheless, this study has some limitations. The sample may not be an accurate reflection of the Chinese population because the included women chose induced abortions in our clinic and received PAC, which may have introduced some bias. In addition, the population of patients attending a tertiary center may not be representative of the entire population.

## 5. Conclusion

In conclusion, the overall number of induced abortions increased in this snapshot of a Chinese population, but the ratio of abortions to live births decreased. However, it must be emphasized that differences in mentality and culture between countries might limit the representativeness of these results.

## Figures and Tables

**Table 1:** Sociodemographic characteristics of women who underwent induced abortions at the Family Planning Clinic of Xiamen Maternity and Child Health Care Hospital in China between 2010 and 2013.

Variables (*n* = 19,655)	Number	%
Age (years, mean = 27 ± 6 years)		
15–19	891	4.5
20–24	6494	33
25–29	6164	31.4
30–34	3690	18.8
35–40	2009	10.2
41–48	407	2.1
Marital status		
Married	11,809	60.1
Unmarried	7846	39.9
Number of pregnancies (including current pregnancy)		
1	5803	29.5
≥2	13,852	70.5
Number of deliveries		
0	9797	49.8
1	8570	43.6
≥2	1288	6.6
Number of abortions (including current abortion)		
1	9300	47.3
2	6173	31.4
≥3	4182	21.3
CS (ever)		
Yes	2576	13.1
No	17,079	86.9
Risk factor(s) present^*∗*^	959	4.9
IUD	349	1.8
Interpregnancy interval of <6 months	391	2
Breastfeeding period	159	0.8
Uterine malformation	77	0.4

CS, caesarian section; IUD, intrauterine device. ^*∗*^Women could have more than one risk factor.

**Table 2:** Ratio of induced abortions to live births between 2010 and 2013.

Year	Induced abortions	Live births	Induced abortions/live births (%)
	*n*	*n*	
2010	3906	8010	48.8
2011	4964	10,211	48.6
2012	5382	12,284	43.4
2013	5403	12,770	42.3

**Table 3:** Trends in the sociodemographic characteristics of women who underwent induced abortions between 2010 and 2013.

Variables	2010	2011	2012	2013	*P*
(*n* = 19,655)	(*n* = 3906)	(*n* = 4964)	(*n* = 5382)	(*n* = 5403)	
Age (years)					
15–24	1659 (42.5)	1958 (39.4)	1979 (36.8)	1789 (33.1)	<0.01
25–34	1787 (45.8)	2415 (48.7)	2740 (50.9)	2912 (53.9)	
34–48	460 (11.8)	591 (11.9)	663 (12.3)	702 (13.0)	
Mean age ± SD	26.71 ± 0.09	26.96 ± 0.08	27.33 ± 0.08	27.70 ± 0.08	
Marital status					
Married	2155 (55.2)	2932 (59.1)	3313 (61.6)	3409 (63.1)	<0.01
Unmarried	1751 (44.8)	2032 (40.9)	2068 (38.4)	1994 (36.1)	
Number of pregnancies					
1	1323 (33.9)	1571 (31.6)	1476 (27.4)	1433 (26.5)	<0.01
≥2	2583 (66.1)	3393 (68.4)	3906 (72.6)	3970 (73.5)	
Number of deliveries					
0	2200 (56.3)	2574 (51.9)	2583 (48.0)	2440 (45.2)	<0.01
1	1529 (39.1)	2114 (42.6)	2443 (45.4)	2484 (46.0)	
≥2	177 (4.5)	276 (5.6)	356 (6.6)	479 (8.9)	
Number of abortions					
1	1929 (49.4)	2436 (49.1)	2413 (44.8)	2522 (46.7)	<0.01
2	1145 (29.3)	1578 (31.8)	1725 (32.1)	1725 (31.9)	
≥3	832 (21.3)	950 (19.1)	1244 (23.1)	1156 (21.4)	
CS					
Yes	371 (9.5)	528 (10.6)	721 (13.4)	957 (17.7)	<0.01
No	3535 (90.5)	4436 (89.4)	4661 (86.6)	4446 (82.3)	
Risk	178 (4.6)	208 (4.2)	256 (4.8)	323 (5.9)	
a	86 (2.20)	95 (1.91)	78 (1.45)	90 (1.67)	
b	54 (1.09)	64 (1.29)	123 (2.29)	151 (2.79)	
c	23 (0.59)	34 (0.68)	47 (0.87)	55 (1.02)	
d	17 (0.44)	16 (0.32)	13 (0.24)	31 (0.57)	

SD, standard deviation; CS, caesarian section; a, IUD; b, interpregnancy interval of <6 months; c, breastfeeding period; d, uterine malformation.
